# Supplementary material for: Monoclonal humanized monovalent antibody blocking therapy for anti-NMDA receptor encephalitis
Source: Nat Commun. 2025 Jun 17;16:5292. doi: 10.1038/s41467-025-60628-1 (PMC12174348; doi:10.1038/s41467-025-60628-1)
Supplement: Supplementary file 1 — Supplementary Information [file 41467_2025_60628_MOESM1_ESM.pdf]

Supplementary Information

Monoclonal humanized monovalent antibody blocking therapy for anti-NMDA receptor encephalitis

Authors:

Atsuo Kanno[1,2†], Takuya Kito[2†], Masashi Maeda[1,2], Shanni Yamaki[1], Yasushi Amano[2], Takuya Shimomura[2], Margarita Anisimova[3], Naomi Kanazawa[4], Koichiro Suzuki[2], Amir Razai[1], Takuma Mihara[2], Kaori Kubo[2], Takeshi Shimada[2], Koji Nakamura[2], Naoko Nomura[2], Yuji Kondo[2], Akira Okimoto[2], Azusa Sugiyama[2], Deborah Park[3], Ivar Stein[3], Samuel Petshow[3], Valentin Vandendoren[5], Sanela Bilic[5], Roghiye Kazimi[1], Vallari Eastman[1], Scott J. Snipas[1], Mathew Mitchell[1], Mari Maurer[1], Marty Jefson[1], Jay Lichter[1], Daisuke Yamajuku[2], Hiroki Shirai[2], Megumi Adachi[2], Daniel J. Hoeppner[2], Satoshi Kubo[1,2], Karen Zito[3], Takahiro Iizuka[4], Peter Flynn[1] and Mitsuyuki Matsumoto[1,2]*

Affiliations:

[1] Arialys Therapeutics, Inc., La Jolla, CA 92037, USA

[2] Astellas Pharma Inc., Tsukuba, Ibaraki 305-8585, Japan

[3] Center for Neuroscience, University of California, Davis, CA 95618, USA

[4] Department of Neurology, Kitasato University School of Medicine, Sagamihara, Kanagawa 252-0374, Japan

[5] Vanadro, LLC, Waukee, IA 50263, USA

† These authors contributed equally to this work.

* To whom correspondence should be addressed:

Mitsuyuki Matsumoto, Ph.D.

Arialys Therapeutics, Inc.

11099 North Torrey Pines Road, Suite 290, La Jolla, CA 92037, USA

Telephone: +1-847-603-4770

Email: mmatsumoto@arialysrx.com

This PDF file includes:

Figures S1-13

Tables S1-10

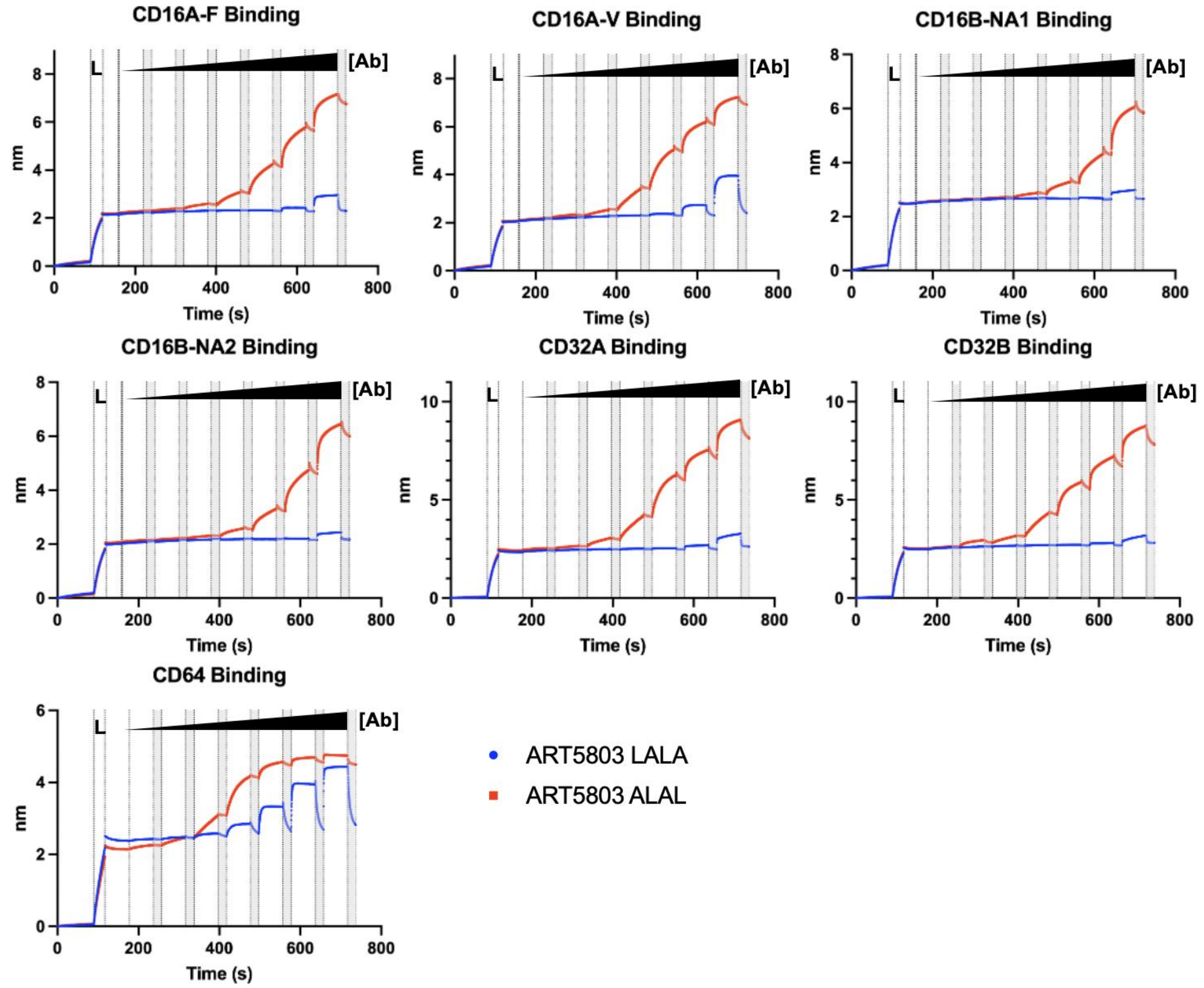

Fig. S1: **ART5803 LALA mutations reduced binding to the Fc receptors.** Biolayer interferometry representing Fc receptor binding of ART5803 (ART5803 LALA) in blue versus an effector enabled wild type version of ART5803 (ART5803 ALAL) in red. Biotinylated Fc receptors were loaded (L) onto streptavidin sensors and observed for binding to increasing amounts (0.64nM, 3.2nM, 16nM, 80nM, 400nM, 2µM, 10µM) of either ART5803 LALA or ART5803 ALAL. A PBS dissociation step (shaded) was included between all association steps. Source data are provided as a Source Data file.

a

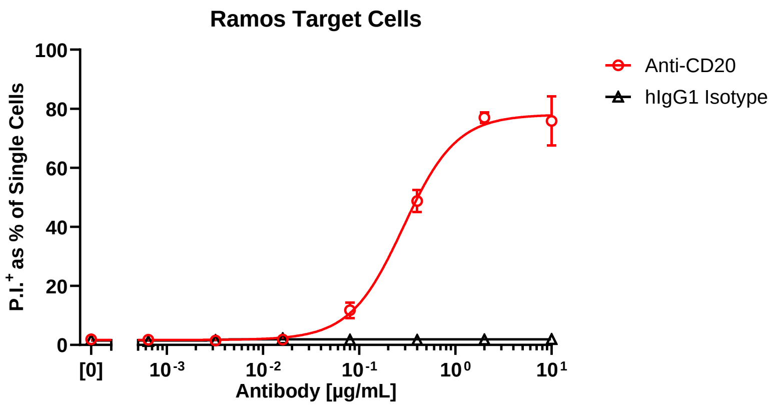

b

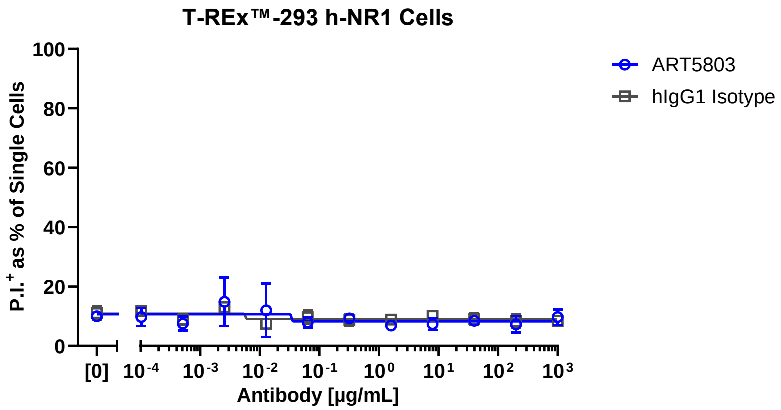

Fig. S2: **ART5803 did not induce CDC on GluN1-expressing cell lines.** Complement-mediated cytotoxicity (CDC) was assessed using propidium iodide (P.I.) incorporation in two different cell lines by flow cytometry. Cells were incubated with serial dilutions of antibody in the presence of 25% (v/v) qualified normal human serum as a source of complement. (a) CDC activity on Ramos target cells treated with anti-CD20 (rituximab, positive control) showed clear dose-dependent cytotoxicity by P.I. incorporation compared to the human IgG1 isotype control treatment. Data are mean ± SD of n=3 internal replicates. (b) CDC activity of ART5803 was evaluated using T-REx™-293 cells stably expressing the human GluN1 subunit of the NMDA receptor (T-REx™-293 h-NR1). ART5803 treatment did not result in elevated P.I. incorporation relative to the isotype control. For both assays, cytotoxicity was quantified as the percentage of P.I. positive cells among single cells. Data are mean ± SD of n=3 internal replicates. These results demonstrate ART5803 does not elicit CDC on GluN1 positive cells, suggesting a favorable safety profile with respect to complement activation. Source data are provided as a Source Data file.

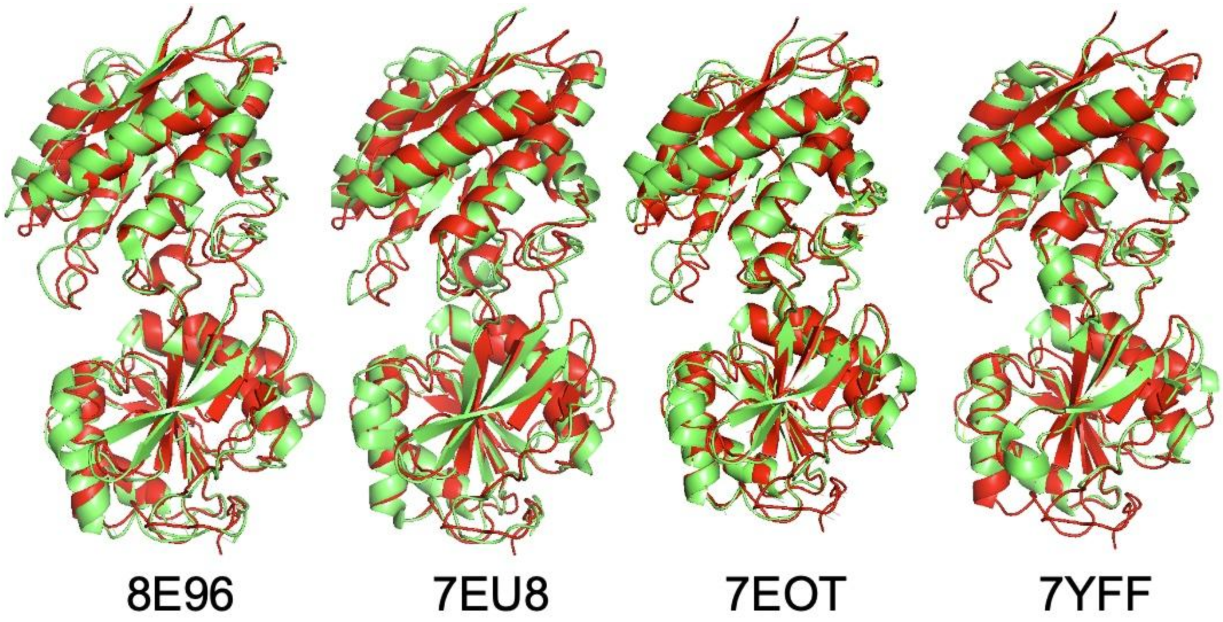

Fig. S3: **Binding to GluN1 by ART5803 epitope competitor #003-102 Ab does not lead to significant conformational change in GluN1.** #003-102 Ab Fab' bound GluN1 NTD (red, #003-102 Ab Fab' structure hidden) aligned against various GluN1 NTD structures reported in various studies across literature (green, #003-102 Ab not bound). PDB ID for the referenced structures are indicated under each structure. 8E96 has NMDAR in its glycine/glutamate bound states, 7EU8 has NMDAR bound to antagonist S-Ketamine and glycine/glutamate, 7EOT has NMDAR with the GluN1 antagonist CGP-78608 and 7YFF has NMDAR with antagonist CPP and glycine/glutamate. In short, #003-102 Ab bound GluN1 NTD (red) was aligned to the four reference structures GluN1 NTDs, (green) separately. Alignments were then visually analyzed for any gross conformational changes between antibody bound (red) and antibody unbound (green) states of GluN1 NTD.

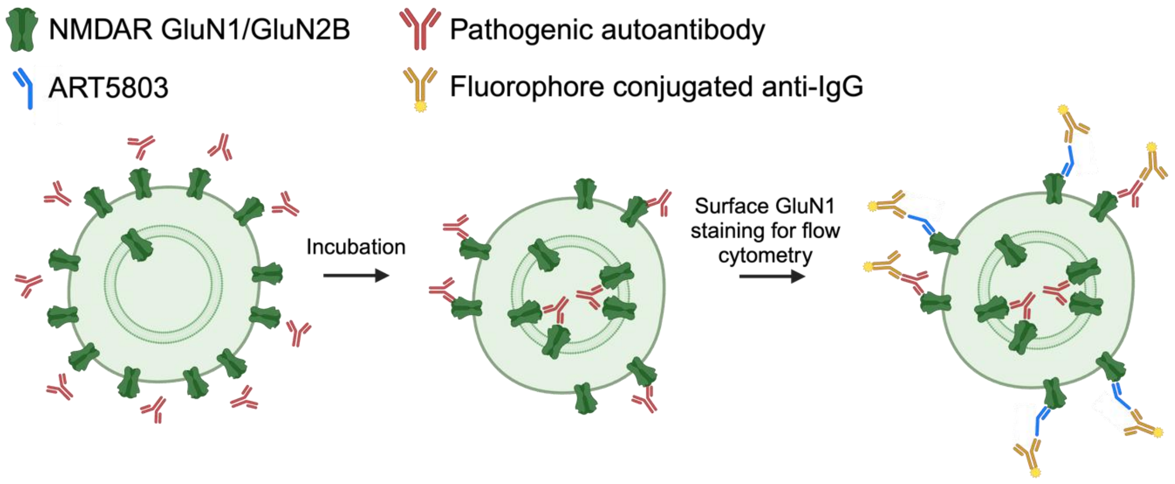

Fig. S4: **Representative experimental design of NMDAR internalization studies using NMDAR-expressing HEK293 cells.** To quantify total NMDAR expression, surface staining is performed with excess ART5803 to ensure all surface NMDARs are occupied by human IgG antibodies for detection. Created in BioRender. Yamaki, S. (2025) https://BioRender.com/k80z736.

a

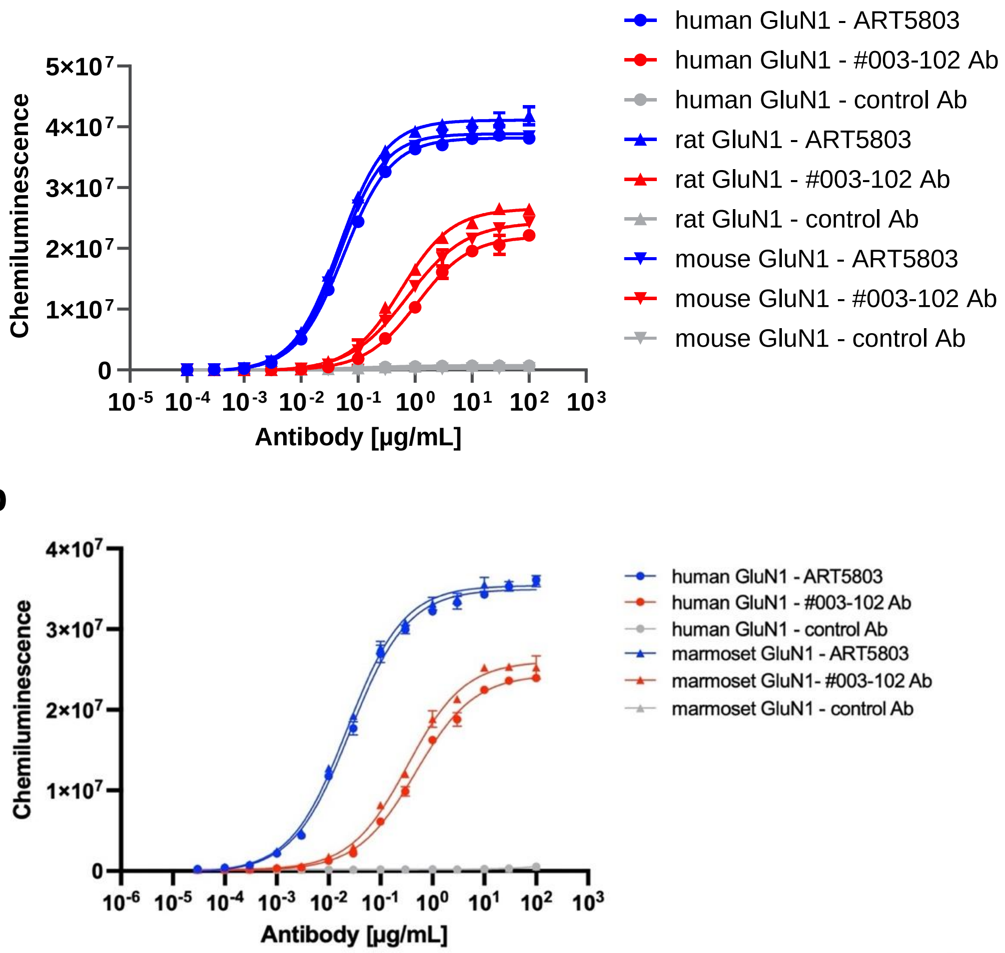

Fig. S5: **Binding activity of ART5803 and #003-102 Ab to GluN1-NTD proteins from human, marmoset, rat, and mouse.** (a) ELISA comparing binding of antibodies ART5803, #003-102 Ab, and human IgG1 isotype control to the NMDAR subunit GluN1 from human, rat, and mouse. Data are mean ± SD of n=2 internal replicates. EC_{50} values for ART5803 binding human, rat, and mouse GluN1 were 0.056, 0.047, 0.048 µg/mL respectively. EC_{50} values for #003-102 Ab binding human, rat, and mouse GluN1 were 1.1, 0.67, 0.74 µg/mL respectively. (b) ELISA comparing binding of antibodies ART5803, #003-102 Ab, and human IgG1 isotype control to the NMDAR subunit GluN1 from human and marmoset. Data are mean ± SD of n=3 internal replicates. EC_{50} values for ART5803 binding human and marmoset NMDAR subunit GluN1 were 0.026 and 0.023 µg/mL respectively. EC_{50} values for #003-102 Ab binding human and marmoset NMDAR subunit GluN1 were 0.47 and 0.33 µg/mL respectively. Source data are provided as a Source Data file.

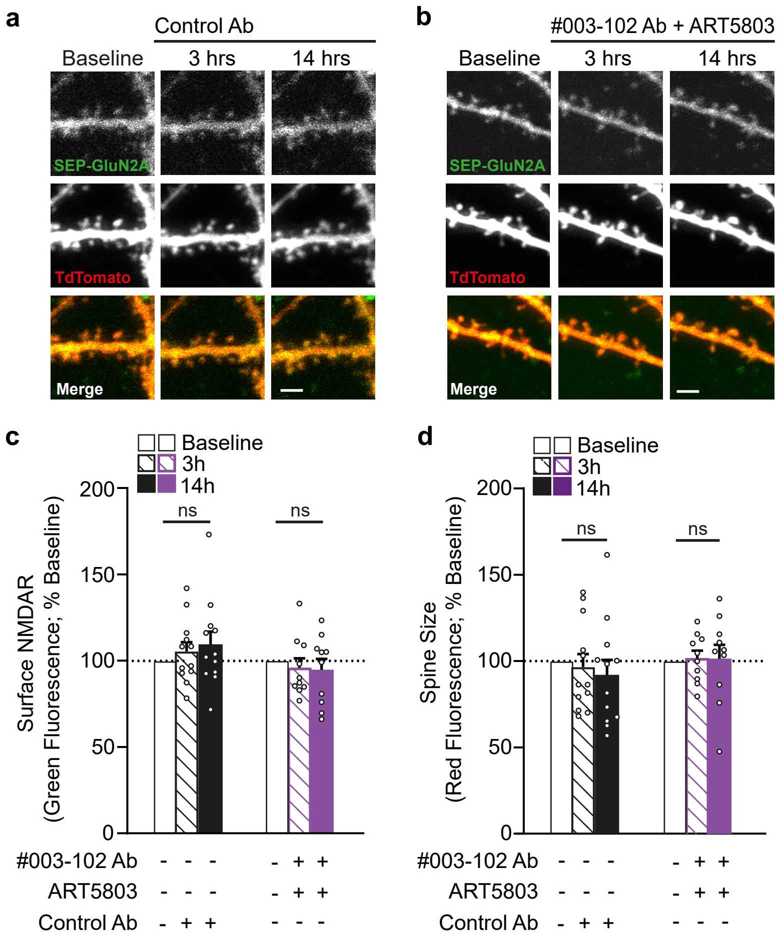

Fig. S6. **Co-incubation of pathogenic #003-102 Ab with ART5803 blocks NMDAR internalization and spine size reduction. (a-b)** Images of dendrites from mouse CA1 pyramidal neurons in hippocampal slice cultures transfected with SEP-GluN2A (green), GluN1 (unlabeled), and tdTomato (red) directly prior to (baseline) and after (3 hours and 14 hours) incubation with (a) control Ab or (b) both pathogenic #003-102 Ab and ART5803. Scale bars are 2 µm. **(c)** Control Ab had no impact on surface NMDAR expression (black; 12 cells). No reduction in surface NMDAR expression was observed when pathogenic #003-102 Ab was mixed with ART5803 (purple; 10 cells). **(d)** Control Ab had no impact on spine size (black; 12 cells). No reduction in spine size was observed when pathogenic #003-102 Ab was mixed with ART5803 (purple; 10 cells). Each point is from an individual spine and bars represent mean ± SEM. Paired ordinary one-way ANOVA with Tukey's multiple comparisons test. Within each condition, no significance (ns) was found for all pairwise comparisons. Source data are provided as a Source Data file.

a

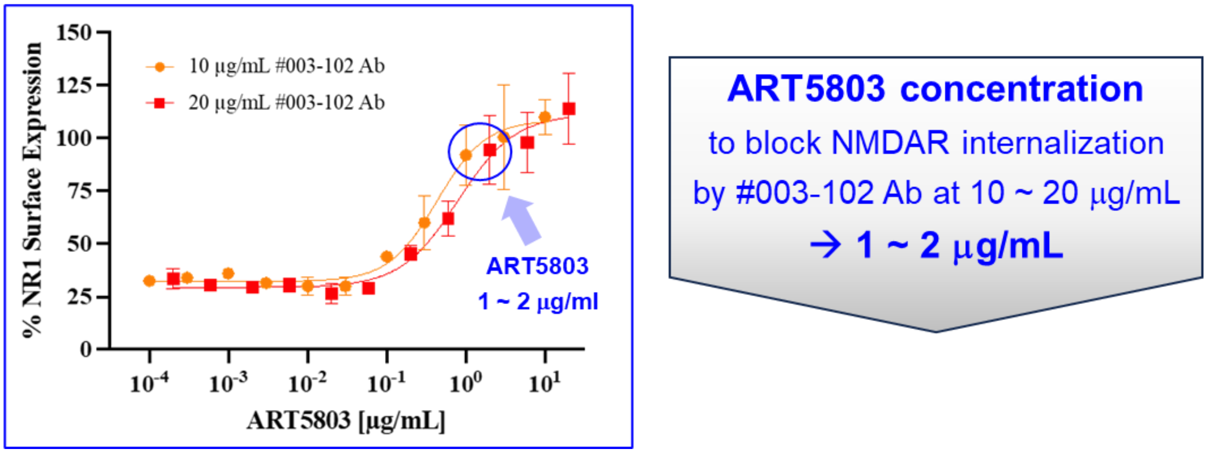

b

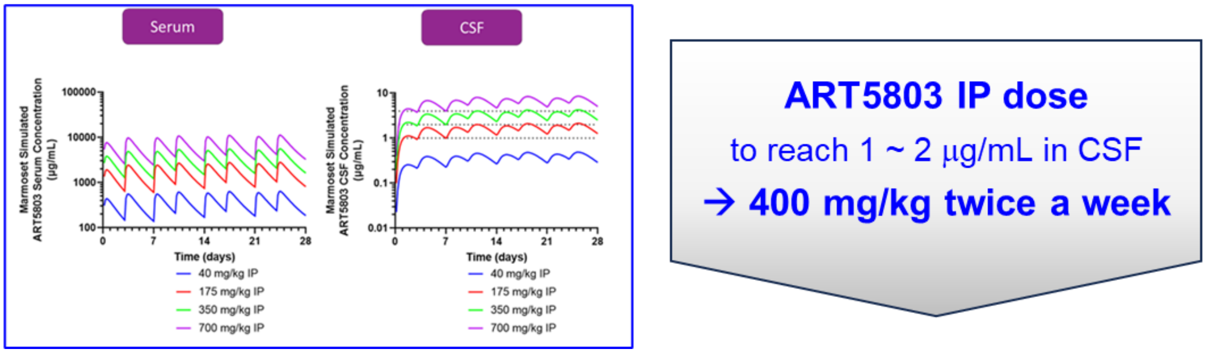

Fig. S7: **Summary of preliminary studies to determine IP dose in marmoset disease model.** (a) HEK293 cells expressing human NMDAR GluN1 and GluN2B subunits were used in a flow cytometry based NMDAR internalization assay to assess the dose dependent ability of ART5803 to block internalization driven by #003-102 Ab. ART5803 was co-incubated overnight with 10 or 20 µg/mL #003-102 Ab. Data are mean of n=2 internal replicates ± SD. (b) The IP dose, 400 mg/kg twice a week, to achieve a target concentration of 1 – 2 µg/mL of ART5803 in the CSF was determined by a preliminary pharmacokinetics (PK) study and simulation in marmosets. Simulations of serum and CSF concentrations are shown. Source data are provided as a Source Data file.

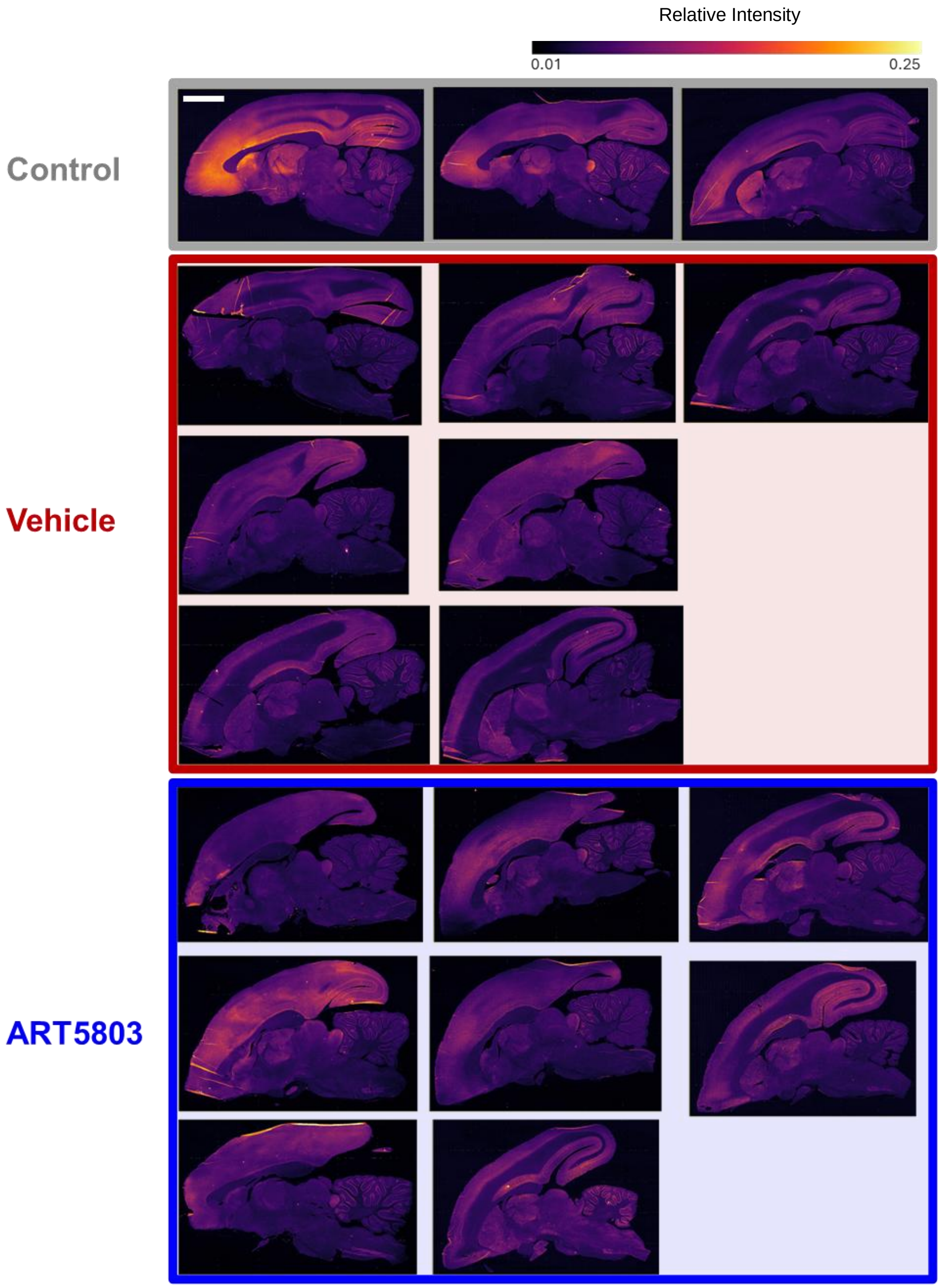

Fig. S8: **Heatmaps of GluN1 staining of marmoset brains after #003-102 Ab ICV + ART5803 or vehicle IP injections.** Average GluN1 DAB mean intensity from superpixels were used to generate heatmaps and displayed as colors ranging from black (less) to yellow (more). The scale bar is 5 mm.

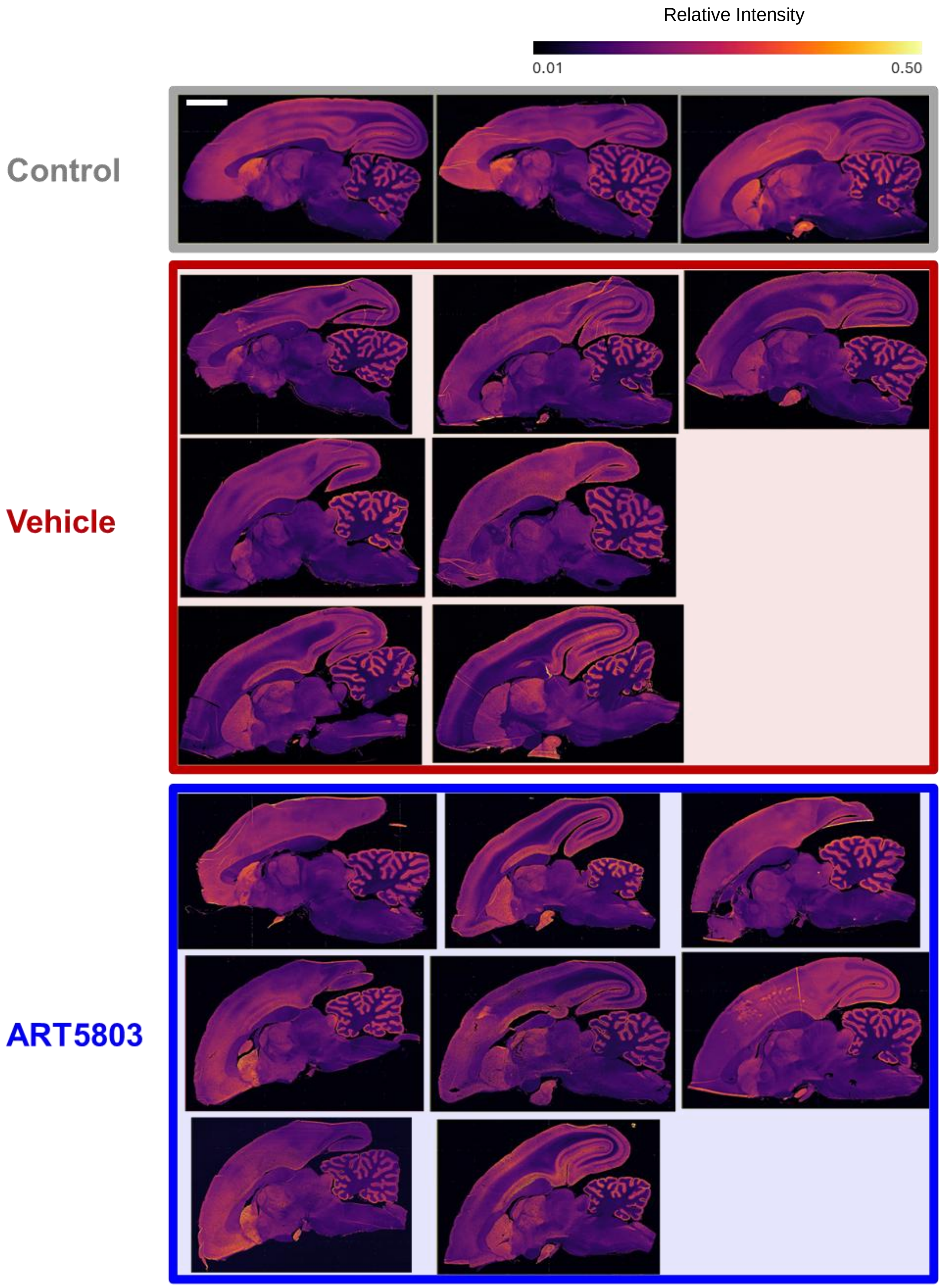

Fig. S9: **Heatmaps of GluR2 staining of marmoset brains after #003-102 Ab ICV + ART5803 or vehicle IP injections.** Average GluR2 DAB mean intensity from superpixels were used to generate heatmaps and displayed as colors ranging from black (less) to yellow (more). The scale bar is 5 mm.

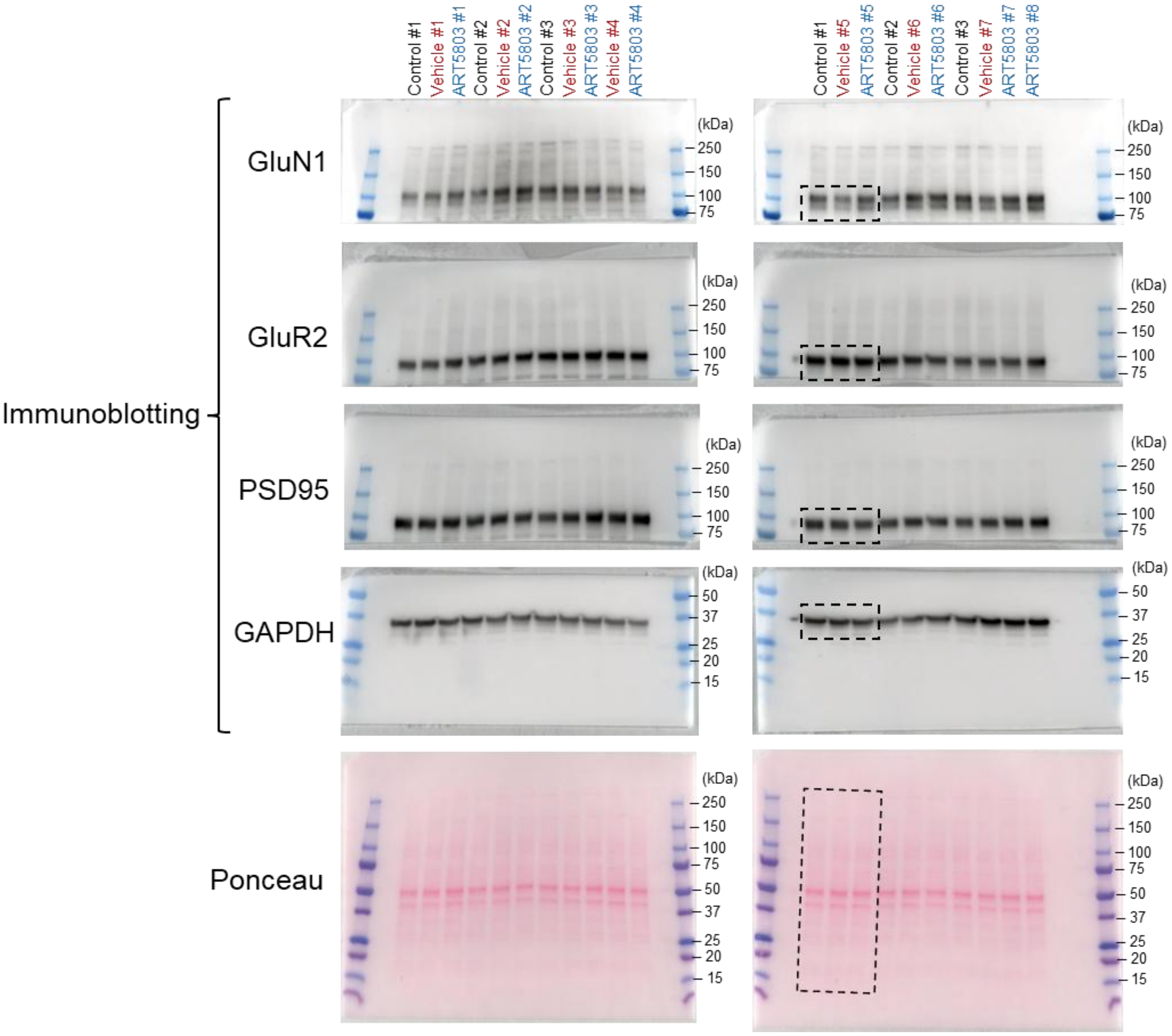

Fig. S10: **Western blot analysis of marmoset brains after #003-102 Ab ICV + ART5803 or vehicle IP injections.** Uncropped scans of the Western blots shown in the indicated figures. Immunoblotting with GluN1, GluR2, PSD95, GAPDH and Ponceau staining using the lysate of marmoset brains (#003-102 Ab ICV + ART5803 or vehicle IP injections).

R2 binding autoantibodies

ART5803 blocks autoantibody binding to the R2 domain by direct epitope competition, preventing NMDARs crosslinking and subsequent internalization.

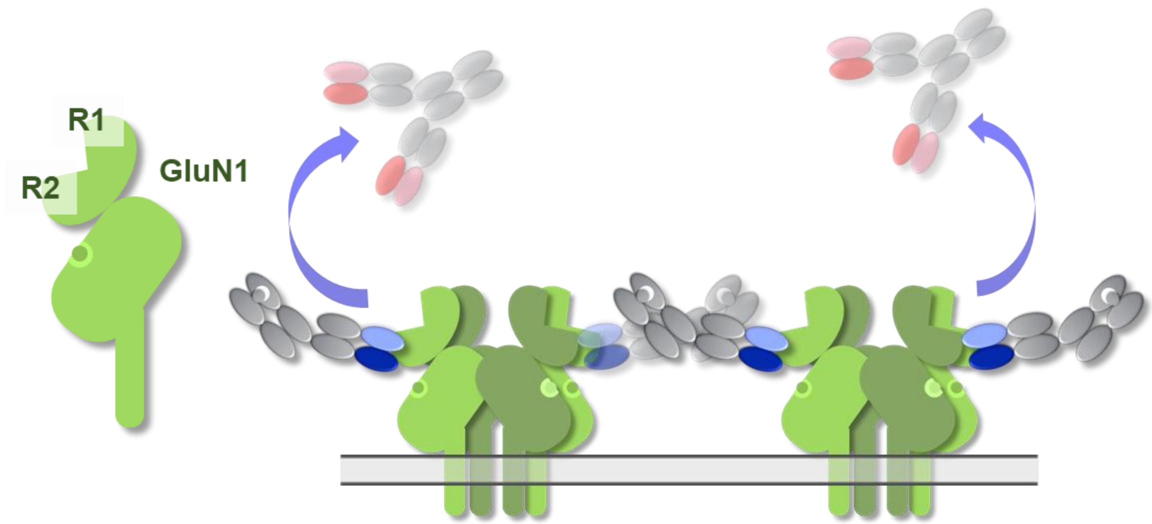

R1 binding autoantibodies

ART5803 blocks autoantibody crosslinking of NMDARs by reinforcing receptor separation. Autoantibodies may bind to one receptor, but ART5803 prevents bridging of two NMDARs through steric hindrance, blocking internalization.

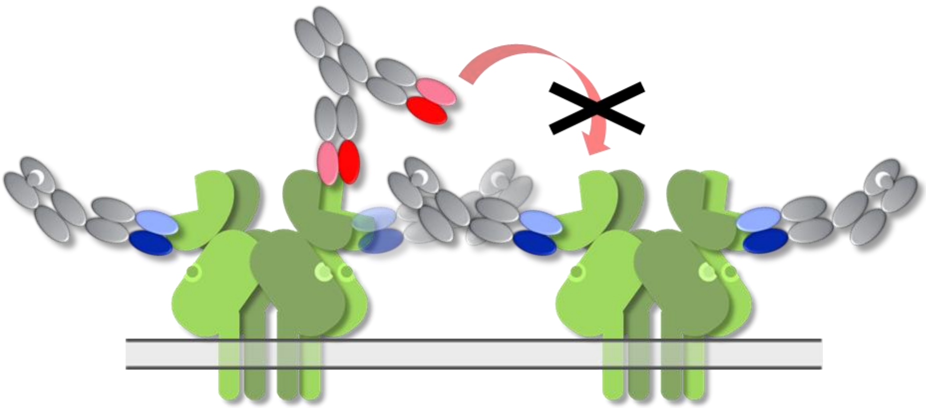

Fig. S11: **Proposed mode of actions of ART5803 against various autoAbs binding to R1 or R2 domains.**

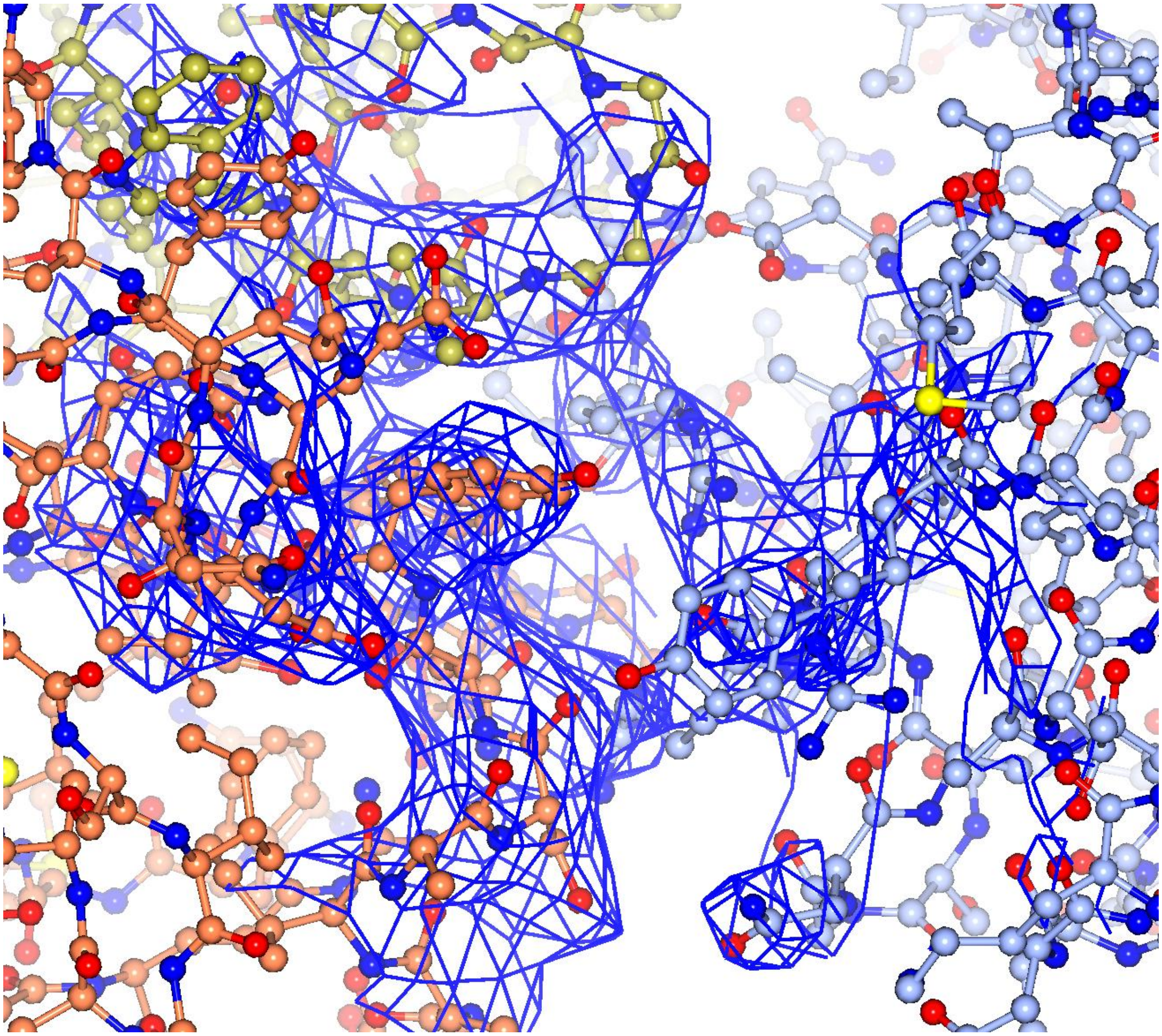

Fig. S12: **Portion of electron density map showing GluN1-NTD and #003-102 Ab Fab' antibody (2Fo−Fc ma, contour level: 1.0σ).** Cyan: GluN1-NTD, Yellow: #003-102 Ab Fab' heavy chain, Orange: #003-102 Ab Fab' light chain.

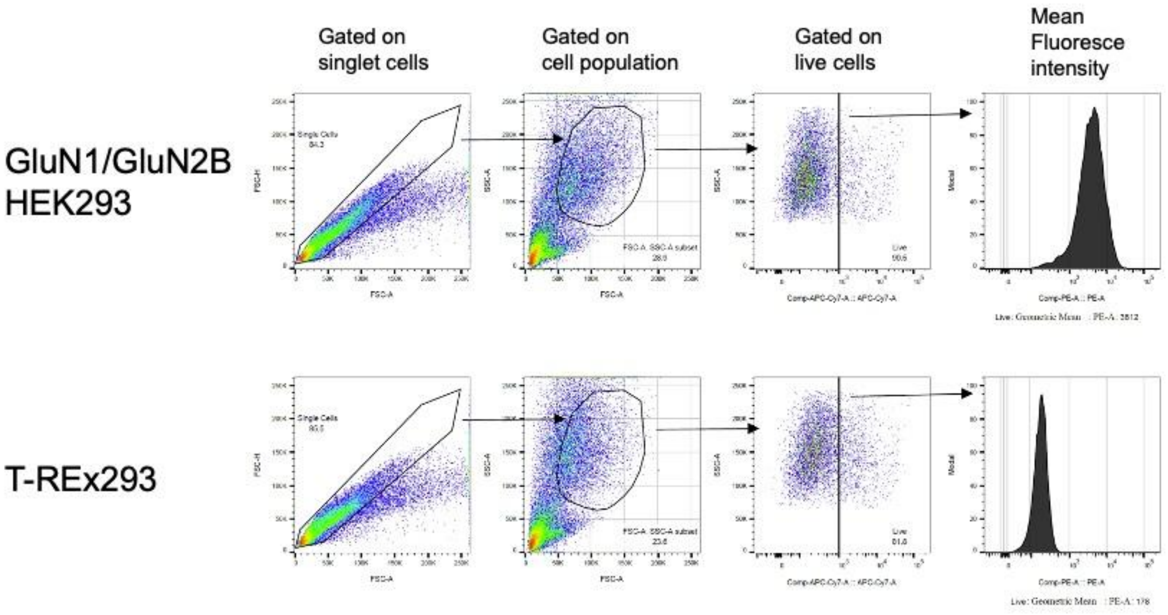

Fig. S13: **Flow cytometry gating strategy for surface GluN1 detection.** Representative staining by using anti-GluN1 human IgG antibody 502 Ab with anti-human IgG PE conjugated antibody on GluN1/GluN2B expressing HEK293 cells or negative control T-REx293 cells.

Table S1. **ART5803 and #003-102 Ab human GluN1 binding affinities (Biacore).**

	**ART5803**	**#003-102 Ab**
k_{on} (M^{-1} s^{-1})	8.83×10^{5}	1.05×10^{4}
k_{off} (s^{-1})	6.11×10^{-4}	3.0×10^{-3}
K_D (M)	6.92×10^{-10}	2.85×10^{-7}

Table S2. **The comparison of epitopes between the results of HDX-MS and X-ray crystallography.**

	HDX-MS		Crystal structure
Region	ART5803	#003-102 Ab	#003-102 Ab
1	K25-V27	K25-V27	
2		K46, K51-H53	
3	V157-N161	V157-N161	
4		A211-A216	
5	E251-L269	E251-L269	N257, R260,Y261, D264
6	N276-S282	N276-S282	
7	Q357-Q363, I366-G391	Q357-Q363, I366-G391	Q357-V362, R377-I380, G383, G384, T386

Amino acids shown in the crystal structure column determined by X-ray crystallography are residues < 4.5 Å from #003-102 Ab.

Table S3. **#003-102 Ab CSF concentrations by 10 μg/hr ICV infusion #003-102 Ab in marmoset CSF**

Animal ID	Sex	Day 14	Day 28
1	F	30.30	16.40
2	F	15.20	4.39
3	F	5.25	12.20
4	F	4.66	12.60
5	M	16.60	14.60
11	F	27.80	14.20
12	F	21.60	NA
15	M	NA	7.99
	Average	17.34	11.77
	SD	10.06	4.18

#003-102 Ab concentrations are in [μg/mL].
SD=Standard Deviation; F=female; M=male; NA=Not applicable

Table S4. **Abnormal behavior scale score (ARS) criteria.**

Parameters		Score	Behavior
Behavior abnormal score	Attention	0	Always responsive to and focused on the object
		1	Sometimes focused on things other than the object
		2	Mostly focused on things other than the object
		3	No focus on the object
	Motivation	0	Immediately moves with interest and maintains interest
		1	Interested, but does not move immediately and/or interest/action are disrupted
		2	Low interest and/or interest/action are significantly disrupted
		3	No interest and no action
	Fear/anxiety	0	Immediately moves with interest and maintains interest
		1	Shows interest and action, but occasionally closes eyes and/or shows escape behavior (e.g. hiding, crouching in the corner)
		2	Shows temporary interest, but gives up on the object and/or shows escape behavior (e.g. hiding, crouching in the corner)
		3	Shows cautious/vigilant behavior
Motor disability score	Speed of movement	0	Normal
		1	Slightly slow
		2	Slow
		3	Very slow
		4	Immobile
	Motor Coordination Agility/rigidity	0	Normal, able to move without difficulty
		1	Able to move, but not quickly and/or smoothly with good balance.
		2	Movement is slow, and only can move when holding onto something. Balance is poor and sometimes falls onto the floor.
		3	Immobile
	Jumping	0	Normal movement by jumping
		1	Hesitates before and/or rarely jumping
		2	Does not jump
	Stereotypy	0	No stereotypy behavior
		1	Observe one type of stereotypy behavior (e.g. itching, blinking, sneezing, head twitching)
		2	Observe two or more stereotypies behaviors (e.g. itching, blinking, sneezing, head twitching)
	Aberrance of limbs	0	Normal
		1	Observe abnormal movements (e.g. convulsion, twitching, shaking, abnormal posture)
		2	No control of limbs

Table S5: **Marmoset total ARS scores after #003-102 Ab ICV + ART5803 or Control Antibody ICV injections.**

	Baseline	#003-102 Ab alone	#003-102 Ab + ART5803 or Control antibody
Animal ID	Day 0	Day 14	Day 28
ART5803-#1	2	7	4
ART5803-#2	2	18	2
ART5803-#3	4	11	10
ART5803-#4	0	5	0
ART5803-#5	2	2	0
ART5803-#6	3	20	6
ART5803-#7	1	13	4
ART5803-#8	1	3	3
ART5803-#9	1	7	3
ART5803 Mean ± SEM	1.8 ± 0.4	9.6 ± 2.1*	3.6 ± 1.0**
Control -#1	3	9	10
Control -#2	0	15	10
Control -#3	0	0	7
Control Mean ± SEM	1.0 ± 1.0	8.0 ± 4.4	9.0 ± 1.0#

SEM=Standard Error Mean

*$p < 0.01$ Statistically significant in comparison to Day 0, by two-tailed, Wilcoxon matched pairs signed rank test

**$p < 0.01$ Statistically significant in comparison to Day 14, by two-tailed, Wilcoxon matched pairs signed rank test

#$p < 0.05$ Statistically significant in comparison to Day 28 ART5803 treated group

Table S6. **IP injections of ART5803 alone twice a week for 2 weeks did not induce any noticeable behavioral changes (clinical signs) in marmosets**

Group	Animal No.	Day (Dosing period)														
		0*	1	2	3*	4	5	6	7*	8	9	10*	11	12	13	14
		Animal Sign														
ART5803	1(m)	(-)	(-)	(-)	(-)	(-)	(-)	(-)	(-)	(-)	(-)	(-)	(-)	(-)	(-)	(-)
200 (mg/kg)	2(f)	(-)	(-)	(-)	(-)	(-)	(-)	(-)	(-)	(-)	(-)	(-)	(-)	(-)	(-)	(-)
	3(f)	(-)	(-)	(-)	(-)	(-)	(-)	(-)	(-)	(-)	(-)	(-)	(-)	(-)	(-)	(-)
ART5803	4(m)	(-)	(-)	(-)	(-)	(-)	(-)	(-)	(-)	(-)	(-)	(-)	(-)	(-)	(-)	(-)
400 (mg/kg)	5(m)	(-)	(-)	(-)	(-)	(-)	(-)	(-)	(-)	(-)	(-)	(-)	(-)	(-)	(-)	(-)
	6(f)	(-)	(-)	(-)	(-)	(-)	(-)	(-)	(-)	(-)	(-)	(-)	(-)	(-)	(-)	(-)
ART5803	7(m)	(-)	(-)	(-)	(-)	(-)	(-)	(-)	(-)	(-)	(-)	(-)	(-)	(-)	(-)	(-)
800 (mg/kg)	8(m)	(-)	(-)	(-)	(-)	(-)	(-)	(-)	(-)	(-)	(-)	(-)	(-)	(-)	(-)	(-)
	9(f)	(-)	(-)	(-)	(-)	(-)	(-)	(-)	(-)	(-)	(-)	(-)	(-)	(-)	(-)	(-)

No.=number, m=male, f=female, *=dosing days, (-)=no behavioral changes

Table S7: **Marmoset total ARS scores after #003-102 Ab ICV + ART5803 or vehicle IP injections.**

Vehicle					ART5803				
Animal No./Day	0	6	14	21	Animal No./Day	0	6	14	21
3^m	2	14	8	12	1^m	0	9	8	7
4^m	3	12	7	9	2^m	4	16	5	8
7^f	1	15	9	10	8^f	3	12	10	12
10^f	4	11	17	14	9^f	3	11^{D7}	4	4
17^m	0	13	14	15	11^f	2	12	7	6
19^f	4	11	12	7	12^f	3	10	5	8
21^f	0	9	13	7	13^m	3	10	9	8
NA	NA	NA	NA	NA	23^f	1	11	10	7
Mean	2.0	$12.1^{\$}$	11.4	10.6	Mean	2.4	$11.4^{\$\$}$	$7.3^{*\#\#}$	$7.5^{*\#}$
SEM	0.7	0.8	1.4	1.2	SEM	0.5	0.8	0.8	0.8

No.=number, m=male, f=female, NA=not applicable, SEM=standard error mean

* p< 0.05 Statistically significant from Vehicle group (t-test)

$ p <0.05 , $$ p <0.01 Statistically significant from day 0 (two-tailed, Wilcoxon matched-pairs signed rank test)

# p <0.05 , ## p <0.01 Statistically significant from day 6 (two-tailed, Wilcoxon matched-pairs signed rank test)

^{D7} Data was obtained from Day 7 and included in this analysis

Table S8a. **Summary Serum Pharmacokinetic Parameters of ART5803 After a Single IV Infusion to Male Cynomolgus Monkeys at Doses of 100 and 1000 mg/kg**

**Matrix**	**Serum**					
**Dose (mg/kg)**	**100 mg/kg**			**1000 mg/kg**		
**Subject**	**N**	**Mean**	**SD**	**N**	**Mean**	**SD**
**C_{max} (μg/mL)**	3	2400	337	3	21000	4250
**t_{max} (hr)***	3	0.083	(0.083-0.5)	3	0.083	(0.083-0.5)
**C_{last} (μg/mL)**	3	0.707	0.451	3	3.44	3
**t_{last} (hr)***	3	2016	(2016-2016)	3	2016	(1344-2016)
**$t_{1/2}$ (hr)**	3	237	2.15	3	220	126
**$AUC_{0-1008h}$ (hr*μg/mL)**	3	175000	28600	3	1150000	93500
**$AUC_{0-1344h}$ (hr*μg/mL)**	3	178000	30300	3	1160000	98800
**AUC_{last} (hr*μg/mL)**	3	179000	31200	3	1160000	103000
**AUC_{inf} (hr*μg/mL)**	3	179000	31300	3	1160000	104000
**AUC %Extrap**	3	0.127	0.0711	3	0.117	0.139
**C_{max}/D (μg/mL/mg)**	3	5.35	0.833	3	4.81	0.711
**$AUC_{0-1344h}$/D (hr*μg/mL/mg)**	3	393	39.7	3	267	14.8
**AUC_{last}/D (hr*μg/mL/mg)**	3	396	41.6	3	268	15.8
**CL (mL/hr)**	3	2.54	0.285	3	3.74	0.221
**Vz (mL)**	3	871	106	3	1160	612

*t_{max} and t_{last} are presented as median and range.

Table S8b. **Summary CSF Pharmacokinetic Parameters of ART5803 After a Single IV Infusion to Male Cynomolgus Monkeys at Doses of 100 and 1000 mg/kg**

Matrix	CSF					
Dose (mg/kg)	100 mg/kg			1000 mg/kg		
Subject	N	Mean	SD	N	Mean	SD
C_{max} (µg/mL)	3	3.01	2.43	3	15.1	8
t_{max} (hr)*	3	24	(24-168)	3	24	(24-24)
C_{last} (µg/mL)	3	0.021	0.0134	3	0.0144	0.0024
t_{last} (hr)*	3	1008	(1008-1344)	3	2016	(1680-2016)
$t_{1/2}$ (hr)	3	208	3.78	3	339	18.1
$AUC_{0-1008h}$ (hr*µg/mL)	3	459	226	3	2190	1020
$AUC_{0-1344h}$ (hr*µg/mL)	3	465	229	3	2210	1030
AUC_{last} (hr*µg/mL)	3	462	227	3	2230	1040
AUC_{inf} (hr*µg/mL)	3	469	231	3	2240	1040
AUC %Extrap	3	1.36	0.417	3	0.343	0.0977
C_{max}/D (µg/mL/mg)	3	0.00671	0.00557	3	0.00342	0.00161
$AUC_{0-1008h}$/D (hr*µg/mL/mg)	3	1.01	0.487	3	0.498	0.203
AUC_{last}/D (hr*µg/mL/mg)	3	1.02	0.488	3	0.507	0.206
CL (mL/hr)	3	1190	712	3	2160	720
Vz (mL)	3	360000	221000	3	1050000	331000

*t_{max} and t_{last} are presented as median and range.

Table S9. **Parasite and bacterial proteins showing 100% match with human NMDAR NR1-NTD peptide LQNRKLV (356-362)**

	Genus	Species	Sequence ID	Protein ID
Parasite	Toxoplasma	Gondii	multi strain (ex. XP_018637765.1)	internal kinesin motor domain protein
Bacteria	Campylobacter	Sonneborni	CAD8123220.1	unnamed protein product
		pelordis	WP_147575871.1	inverse autotransporter beta domain-containing protein
		ornithocola	QKF57284.1	hypothetical protein CORN_0763
			WP_066008439.1	tetratricopeptide repeat protein
		lari	WP_257397452.1	hypothetical protein
			EAK0494263.1	hypothetical protein
	Enterococcus	caccae	WP_010772549.1	DNA primase
	Peptoniphilus	ING2-D1G	CDZ75713.1	hemolysin
		catoniae	WP_138160600.1	MalY/PatB family protein
	Paenalcaligenes	suwonensis	WP_166409601.1	LysR family transcriptional regulator
	Pseudomonas	multispecies	MAP30115.1	2,5-didehydrogluconate reductase DkgB
		viridiflava	WP_122808001.1	PAS domain-containing methyl-accepting chemotaxis protein
		mendocina NK-01	AEB56118.1	aldo/keto reductase
	Burkholderia	multispecies	WP_166913548.1	2,5-didehydrogluconate reductase DkgB
		multivorans CGD2	EEE05529.1	2,5-diketo-D-gluconic acid reductase A
		multivorans CGD1	EEE01682.1	2,5-diketo-D-gluconic acid reductase B
		multivorans CGD2	EEE05529.1	
		cenocepacia K56-2Valvano	EPZ89645.1	
		diffusa	CAG9252122.1	methylglyoxal reductase DkgB
		cenocepacia	CAD9221280.1	
		cenocepacia	SOT44821.1	Aldehyde reductase
		dolosa AU0158	EAY71159.1	NADP dependent sorbitol 6-phosphate dehydrogenase
		multivorans CF2	EJO61145.1	oxidoreductase, aldo/keto reductase family protein

Table S9 (continued). **Parasite and bacterial proteins showing 100% match with human NMDAR NR1-NTD peptide LQNRKLV (356-362)**

	Genus	Species	Sequence ID	Protein ID
Bacteria	Chromobacterium	multispecies	WP_118268538.1	2,5-didehydrogluconate reductase DkgB
	Acinetobacter	baumannii	WP_114159739.1	LysR family transcriptional regulator
		calcoaceticus/baumanni complex	WP_001163442.1	
		nosocomialis	WP_104918929.1	
		baumannii	WP_162283112.1	LysR substrate-binding domain-containing protein
		A47	WP_038346411.1	2,5-didehydrogluconate reductase DkgB
		S40	WP_200458926.1	
		unclassified, multispecies	WP_200482793.1	
		ANC3789	WP_004749200.1	
	Paenibacillus	physcomitrellae	WP_094095067.1	Gfo/Idh/MocA family oxidoreductase
	Escherichia	coli	EFA4879647.1	EscU/YscU/HrcU family type III secretion export apparatus switch protein
		marmotae	WP_038355821.1	
		coli	WP_224219376.1	2,5-didehydrogluconate reductase DkgB
		coli	EET3164582.1	
	Nocardia	multispecies	WP_040703631.1	hypothetical protein

Table S10. **Data collection and refinement statistics (molecular replacement).**

	GluN1-NTD / #003-102 Ab Fab'
**Data collection**	
Space group	$P3_1$
Cell dimensions	
a, *b*, *c* (Å)	287.01, 287.01, 53.87
α, β, γ (°)	90, 90, 120
Resolution (Å)	49.71 – 3.50 (3.59 – 3.50) *
R_{merge}	0.268 (2.794)
$I / \sigma I$	16.03 (1.81)
Completeness (%)	100.0 (100.0)
Redundancy	42.3 (36.6)
**Refinement**	
Resolution (Å)	49.71 – 3.50
No. reflections	59463
R_{work} / R_{free}	0.186 / 0.227
No. atoms	
Protein	24484
B-factors	
Protein	132.166
R.m.s. deviations	
Bond lengths (Å)	0.012
Bond angles (°)	1.664

*Values in parentheses are for highest-resolution shell.
