# Supplementary material for: Monoclonal humanized monovalent antibody blocking therapy for anti-NMDA receptor encephalitis
Source: Nat Commun. 2025 Jun 17;16:5292. doi: 10.1038/s41467-025-60628-1 (PMC12174348; doi:10.1038/s41467-025-60628-1)
Supplement: Supplementary file 2 — Description of Additional Supplementary Files [file 41467_2025_60628_MOESM2_ESM.docx]

**Legends for Supplementary Movies**

**Supplementary Movie 1**

Normal marmoset behavior in response to treats on Day 0, prior to ICV infusion of pathogenic antibody #003-102 Ab.

**Supplementary Movie 2**

Abnormal marmoset behavior in response to treats on Day 14, following two weeks of ICV infusion with pathogenic antibody #003-102 Ab, prior to ART5803 ICV administration.

**Supplementary Movie 3**

Restored normal marmoset behavior in response to treats on Day 15, 1 day after ICV administration of ART5803 in the presence of ongoing pathogenic antibody #003-102 Ab ICV infusion. Please note that this marmoset was a rapid responder to ART5803 treatment.

**Supplementary Movie 4**

Normal marmoset behavior during interaction with caregiver on Day 0, prior to ICV infusion of pathogenic antibody #003-102 Ab.

**Supplementary Movie 5**

Abnormal marmoset behavior during interaction with caregiver on Day 7, after 1 week of ICV infusion with pathogenic antibody #003-102 Ab, prior to ART5803 IP injections.

**Supplementary Movie 6**

Restored normal marmoset behavior during interaction with caregiver on Day 14, after two ART5803 IP injections for 1 week in the presence of ongoing pathogenic antibody #003-102 Ab ICV infusion.
