# Supplementary material for: Monoclonal humanized monovalent antibody blocking therapy for anti-NMDA receptor encephalitis
Source: Nat Commun. 2025 Jun 17;16:5292. doi: 10.1038/s41467-025-60628-1 (PMC12174348; doi:10.1038/s41467-025-60628-1)
Supplement: Supplementary file 9 — Reporting Summary [file 41467_2025_60628_MOESM9_ESM.pdf]

nature portfolio

Corresponding author(s): Mitsuyuki MATSUMOTO, PhD.

Last updated by author(s): May 20, 2025

Reporting Summary

Nature Portfolio wishes to improve the reproducibility of the work that we publish. This form provides structure for consistency and transparency in reporting. For further information on Nature Portfolio policies, see our Editorial Policies and the Editorial Policy Checklist.

Statistics

For all statistical analyses, confirm that the following items are present in the figure legend, table legend, main text, or Methods section.

n/a	Confirmed	
[ ]	[x]	The exact sample size (*n*) for each experimental group/condition, given as a discrete number and unit of measurement
[ ]	[x]	A statement on whether measurements were taken from distinct samples or whether the same sample was measured repeatedly
[ ]	[x]	The statistical test(s) used AND whether they are one- or two-sided<br>*Only common tests should be described solely by name; describe more complex techniques in the Methods section.*
[x]	[ ]	A description of all covariates tested
[x]	[ ]	A description of any assumptions or corrections, such as tests of normality and adjustment for multiple comparisons
[ ]	[x]	A full description of the statistical parameters including central tendency (e.g. means) or other basic estimates (e.g. regression coefficient) AND variation (e.g. standard deviation) or associated estimates of uncertainty (e.g. confidence intervals)
[ ]	[x]	For null hypothesis testing, the test statistic (e.g. *F*, *t*, *r*) with confidence intervals, effect sizes, degrees of freedom and *P* value noted<br>*Give P values as exact values whenever suitable.*
[x]	[ ]	For Bayesian analysis, information on the choice of priors and Markov chain Monte Carlo settings
[x]	[ ]	For hierarchical and complex designs, identification of the appropriate level for tests and full reporting of outcomes
[ ]	[x]	Estimates of effect sizes (e.g. Cohen's *d*, Pearson's *r*), indicating how they were calculated

Our web collection on statistics for biologists contains articles on many of the points above.

Software and code

Policy information about availability of computer code

Data collection	X-ray data was collected at SPring-8 BL-32XU
Data analysis	X-ray data was analyzed using:<br>XDS (version Jan 26, 2018 BUILT=20180808), KAMO<br>CCP4 suite (Phaser, REFMAC5(version 5.8.0135))<br>Coot was used for modelling.<br>PyMol Molecular Graphics System was used to generate figure images of determined structures.<br>Antigen-antibody complexes were analyzed using DynamX software in HDX-MS.<br>BIAevaluation software was used in Biacore.<br>QuPath (Version5.1) was used in IHC analysis.<br>ImageJ (version 1.54g) was used in the quantification of Western Blotting.<br>FlowJo (version 10.6.1 and 10.7.2) was used in Flow cytometry analysis.<br>Statistical analysis of assays were analyzed using GraphPad Prism (version 5-9,10).<br>All references are given in the Material and Methods section.

For manuscripts utilizing custom algorithms or software that are central to the research but not yet described in published literature, software must be made available to editors and reviewers. We strongly encourage code deposition in a community repository (e.g. GitHub). See the Nature Portfolio guidelines for submitting code & software for further information.

Data

Policy information about availability of data

All manuscripts must include a data availability statement. This statement should provide the following information, where applicable:

- Accession codes, unique identifiers, or web links for publicly available datasets
- A description of any restrictions on data availability
- For clinical datasets or third party data, please ensure that the statement adheres to our policy

The crystal structure data in this study have been deposited in the Protein Data Bank under accession code 8ZH7.
The data that support the findings are available within the article, supplementary data files and source data.

Research involving human participants, their data, or biological material

Policy information about studies with human participants or human data. See also policy information about sex, gender (identity/presentation), and sexual orientation and race, ethnicity and racism.

Reporting on sex and gender	This study utilized serum and CSF samples collected from two men and five women (aged 14-45 years) diagnosed with NMDAR encephalitis.
Reporting on race, ethnicity, or other socially relevant groupings	The human serum and cerebrospinal fluid samples used in this study were Asian.
Population characteristics	Patients diagnosed with NMDAR encephalitis
Recruitment	Patients who tested positive for anti-NMDAR antibodies were selected.
Ethics oversight	All experiments with human samples were approved by the Health Research Ethics Committee of Kitasato University (approval #C18-297) and/or Astellas Research Ethics Committee (approval #A190154).

Note that full information on the approval of the study protocol must also be provided in the manuscript.

Field-specific reporting

Please select the one below that is the best fit for your research. If you are not sure, read the appropriate sections before making your selection.

[x] Life sciences [ ] Behavioural & social sciences [ ] Ecological, evolutionary & environmental sciences

For a reference copy of the document with all sections, see nature.com/documents/nr-reporting-summary-flat.pdf

Life sciences study design

All studies must disclose on these points even when the disclosure is negative.

Sample size	Sample size was chosen based on preliminary studies.
Data exclusions	No data exclusion.
Replication	2 or more independent replicates was included in vitro studies to confirm results except for human sample (serum/CSF) studies where limitation of sample volumes allowed us to conduct only one-time study.
Randomization	In all animal experiments, the allocation into experimental groups was random.
Blinding	Time-lapse two-photon imaging were analyzed blind to treatment conditions.<br>Marmoset behavioral observation were conducted blind when reviewing the video recordings.<br>Binding was not performed in other experiments because blinding was not a relevant feature.

Reporting for specific materials, systems and methods

We require information from authors about some types of materials, experimental systems and methods used in many studies. Here, indicate whether each material, system or method listed is relevant to your study. If you are not sure if a list item applies to your research, read the appropriate section before selecting a response.

Materials & experimental systems

n/a	Involved in the study
☐	☒ Antibodies
☐	☒ Eukaryotic cell lines
☒	☐ Palaeontology and archaeology
☐	☒ Animals and other organisms
☒	☐ Clinical data
☒	☐ Dual use research of concern
☒	☐ Plants

Methods

n/a	Involved in the study
☒	☐ ChIP-seq
☐	☒ Flow cytometry
☒	☐ MRI-based neuroimaging

Antibodies

Antibodies used

Therapeutic antibody ART5803
Pathogenic anti-NMDAR autoantibodies (clones; #003-102 Ab, #007-124 Ab, #007-168 Ab, #007-169 Ab, #008-218 Ab)
Anti-keyhole limpet hemocyanin (KLH) antibody (isotype control human IgG1)
Reference pathogenic anti-NMDAR autoantibody 502 Ab.

Commercial antibodies
1. Human IgG isotype control antibody (BioLegend, Cat# 403502)
2. APC-conjugated goat anti-human IgG (Jackson ImmunoResearch, Cat# 109-136-170, 1:100 dilution)
3. PE-conjugated goat anti-human IgG (Jackson ImmunoResearch, Cat# 109-116-098, 1:100 dilution)
4. Fc Receptor Binding Inhibitor Polyclonal Antibody (Invitrogen Cat#14-9161-73m, 1:50 dilution)
5. High Sensitivity Streptavidin-HRP (ThermoFisherScientific Cat#21130, 1:8000 dilution)

Validation

Therapeutic antibody ART5803, pathogenic anti-NMDAR autoantibodies (clones; #003-102 Ab, #007-124 Ab, #007-168 Ab, #007-169 Ab, #008-218 Ab), anti-keyhole limpet hemocyanin (KLH) antibody (isotype control human IgG1), and reference pathogenic anti-NMDAR autoantibody 502 Ab were developed by Astellas Pharma Inc. and validated in this study.

Other antibodies were bought from commercial vendors and were validated by the manufacturers.
1. Human IgG isotype control antibody (https://www.biolegend.com/ja-jp/explore-new-products/ultra-leaf-purified-human-igg1-isotype-control-recombinant-antibody-14241?GroupID=GROUP117)
2. APC-conjugated goat anti-human IgG (https://www.jacksonimmuno.com/catalog/products/109-136-170)
3. PE-conjugated goat anti-human IgG (https://www.jacksonimmuno.com/catalog/products/109-116-098)
4. Fc Receptor Binding Inhibitor Polyclonal Antibody (https://www.thermofisher.com/antibody/product/Fc-Receptor-Binding-Inhibitor-Antibody-Polyclonal/14-9161-73)
5. High Sensitivity Streptavidin-HRP (https://www.thermofisher.com/order/catalog/product/21130?SID=srch-srp-21130)

Eukaryotic cell lines

Policy information about cell lines and Sex and Gender in Research

Cell line source(s)

We purchased the Human NR1/2B expressing HEK293 cell line from Charles River (Catalog #CTN6121). We purchased the T-RExTM-293 cell line from Invitrogen (Catalog #R71007). We purchased the ExpiCHO-S (Catalog #A29127), Expi293F (Catalog #A14527), and FreeStyleTM 293-F Cells (Catalog # R79007) from Gibco. CHOK1SV cell line were provided by Lonza Biologics.

Authentication

All cell lines were authenticated by the manufacturer and can be found here:
Human NR1/2B expressing HEK293: https://www.criver.com/cell-lines/human-nmda-nr1nr2b-receptor-cell-line
T-RExTM-293: https://www.thermofisher.com/order/catalog/product/R71007
ExpiCHO-S: https://www.thermofisher.com/order/catalog/product/A29127?SID=srch-srp-A29127
Expi293F: https://www.thermofisher.com/order/catalog/product/A14527
CHOK1SV : https://www.lonza.com/knowledge-center/biologics/w/chok1sv-gs-ko-cell-line
FreeStyleTM293-F Cells: https://www.thermofisher.com/order/catalog/product/R79007?SID=srch-srp-R79007

Mycoplasma contamination

All cell lines were tested negative for mycoplasma by the manufacturer.

Commonly misidentified lines
(See ICLAC register)

No commonly misidentified lines were used.

Animals and other research organisms

Policy information about studies involving animals; ARRIVE guidelines recommended for reporting animal research, and Sex and Gender in Research

Laboratory animals

Male and female wild type mice (C57BL/6J) were purchased from Jackson Laboratory (Bar Harbor, Maine), Envigo (Cumberland,

	Virginia), or bred in-house (University of California Davis, Davis, CA). Adult common marmosets (12 males, more than 1 year old) for ICV infusion and adult common marmosets (15 males and females, more than 1 year old) were purchased from Shin Nippon Biomedical Laboratories, LTD (Kagoshima, Japan). Cynomolgus monkeys (6 males more than 1 year old) were subjected to PK evaluation at Laboratory Corporation of America Holdings (Labcorp).
Wild animals	Please see "Laboratory animals" above.
Reporting on sex	Tests based on sex differences were not conducted. Any differences related to sex were not analyzed.
Field-collected samples	Organotypic hippocampal slice cultures were prepared from postnatal day 7-8 C57BL/6J wild-type mice of both sexes. Plasma prepared from the brain, cerebrospinal fluid, and peripheral blood of marmosets was collected on the final day of the experiment.
Ethics oversight	For ICV infusion evaluation, marmoset procedures in the Protocol were in compliance with applicable animal welfare acts and were approved by the Institutional Animal Care and Use Committee (IACUC) of Astellas Pharma Inc. (Tokyo, Japan) .<br>For IP injection evaluation, marmoset procedures in the Protocol were in compliance with applicable animal welfare acts and were approved by the Institutional Animal Care and Use Committee (IACUC) of SNBL (Kagoshima, Japan).<br>All mouse experimental protocols were approved by the University of California Davis Animal Care and Use Committee.<br>PK assessment in Cynomolgus monkeys was conducted at Labcorp. All procedures in the protocol were in compliance with applicable animal welfare acts and were approved by the local Institutional Animal Care and Use Committee.

Note that full information on the approval of the study protocol must also be provided in the manuscript.

Plants

Seed stocks	N/A
Novel plant genotypes	N/A
Authentication	N/A

Flow Cytometry

Plots

Confirm that:

- [ ] The axis labels state the marker and fluorochrome used (e.g. CD4-FITC).
- [x] The axis scales are clearly visible. Include numbers along axes only for bottom left plot of group (a 'group' is an analysis of identical markers).
- [ ] All plots are contour plots with outliers or pseudocolor plots.
- [ ] A numerical value for number of cells or percentage (with statistics) is provided.

Methodology

Sample preparation	Effects of ART5803 only on internalization, Effects of #003-102 Ab only on internalization, ART5803 block of #003-102 Ab internalization, and ART5803 rescue of #003-102 Ab internalization:<br>All steps were performed on ice or at 4˚C. Cells were disassociated by cell dissociation buffer, enzyme- free (Gibco). Cells were incubated with human Fc receptor binding inhibitor (Invitrogen) and LIVE/DEAD fixable Violet viability kit for 15 minutes (Invitrogen). Cells were washed twice with flow cytometry buffer (2% FBS,1xPBS), and fixed with 4% PFA in PBS for 20 minutes. Cells were resuspended in flow cytometry buffer and stored overnight at 4˚C. Cells were incubated for 30 minutes in primary antibody (ART5803 5 µg/mL or human IgG1 isotype control 5 µg/mL (BioLegend)). Cells were washed twice with flow cytometry buffer. Secondary antibody goat anti-human IgG APC (1:100, Jackson ImmunoResearch) was incubated for 30 minutes protected from light. Cells were then washed twice with flow cytometry buffer and resuspended in flow cytometry buffer for analysis.<br><br>ART5803 block or rescue of monoclonal antibody mixture induced internalization:<br>All steps were performed on ice or at 4˚C. Cells were disassociated by cell dissociation buffer, enzyme- free (Gibco) and washed once with flow cytometry buffer (2% FBS, 1xPBS). Cells were incubated with human Fc receptor binding inhibitor (Invitrogen), then incubated for 30 minutes in primary antibody (ART5803 5 µg/mL or anti-KLH human IgG1 Isotype control 5 µg/mL). Cells were then washed once with flow cytometry buffer. Secondary antibody goat anti-human IgG PE or APC (1:100, Jackson ImmunoResearch) was incubated for 30 minutes protected from light. Cells were then washed once flow cytometry buffer and resuspended in flow cytometry buffer for analysis.<br><br>Detection of autoantibody induced internalization in patient serum and CSF:<br>All steps were performed on ice or at 4˚C. Cells were disassociated by cell dissociation buffer, enzyme- free (Gibco) and

incubated with human Fc receptor binding inhibitor (Invitrogen) and LIVE/DEAD fixable near-IR viability kit for 15 minutes (Invitrogen). Then cells were incubated for 30 minutes in primary antibody (502 Ab 5 µg/mL or anti-KLH human IgG1 isotype control 5 µg/mL). After one wash flow cytometry buffer (2% FBS, 1xPBS), the cells were incubated for 30 minutes with goat anti-human IgG PE (1:100, Jackson ImmunoResearch). After two washes with flow cytometry buffer, cells were fixed with 4% PFA in 1x PBS for 20 minutes then analyzed.

Detection of autoantibodies in patient serum and CSF:
All steps were performed on ice or at 4˚C. Cells were disassociated by cell dissociation buffer, enzyme- free (Gibco). Patient sample were diluted to 1% (serum) and 40% (CSF) and incubated with cells for 30 minutes. After one wash flow cytometry buffer (2% FBS, 1xPBS), cells were incubated with goat anti-human IgG PE (1:100, Jackson ImmunoResearch) for 30 minutes protected from light. Cells were then washed twice with flow cytometry buffer and fixed with 4% PFA in 1x PBS for 20 minutes then analyzed.

Instrument	Cells were analyzed by using FACS Verse (BD) and Novocyte 2060 (Agilent).
Software	Data collection was performed using FACSDiva (BD) and NovoExpress (Agilent). Data was analyzed with FlowJo software (BD).
Cell population abundance	At least 2,000-25,000 cells were acquired for each condition.
Gating strategy	Refer to Supplemental Figure 4 for gating strategy.

☒ Tick this box to confirm that a figure exemplifying the gating strategy is provided in the Supplementary Information.
